# Toward Efficient Synthesis of Porous All-Carbon-Based Nanocomposites for Enantiospecific Separation

**DOI:** 10.1021/acsami.1c02673

**Published:** 2021-05-12

**Authors:** Milena Perovic, Sapir Shekef Aloni, Wuyong Zhang, Yitzhak Mastai, Markus Antonietti, Martin Oschatz

**Affiliations:** † Department of Colloid Chemistry, Max-Planck Institute of Colloids and Interfaces, Am Mühlenberg 1, 14476 Potsdam, Germany; ‡ Department of Chemistry and the Institute of Nanotechnology, Bar-Ilan University, Ramat-Gan 5290002, Israel; § Institute for Technical Chemistry and Environmental Chemistry, Center for Energy and Environmental Chemistry Jena (CEEC Jena), Friedrich-Schiller-University Jena, Philosophenweg 7a, 07743 Jena, Germany

**Keywords:** chiral separation, ionic liquid, enantioselective seperation, porous carbon, chiral carbon, chiral composite

## Abstract

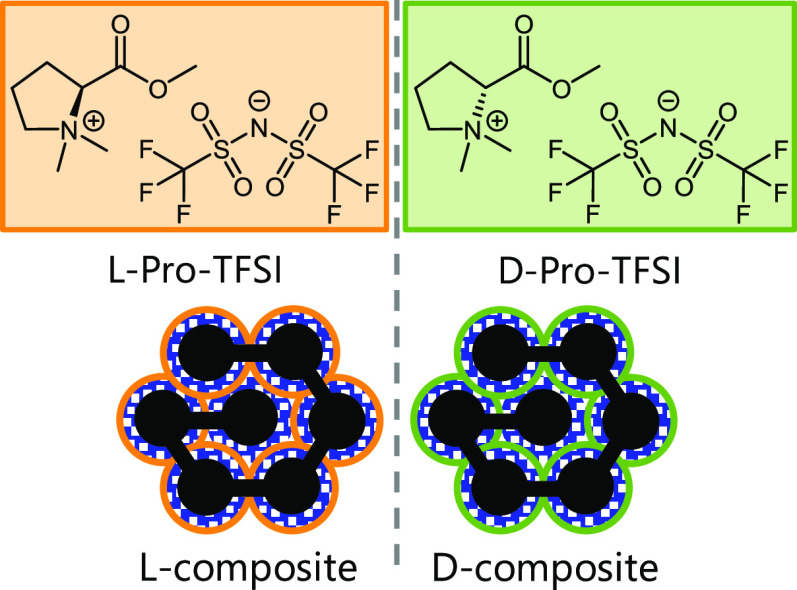

Chiral separation and asymmetric synthesis and catalysis are crucial processes for obtaining enantiopure compounds, which are especially important in the pharmaceutical industry. The efficiency of the separation processes is readily increased by using porous materials as the active material can interact with a larger surface area. Silica, metal–organic frameworks, or chiral polymers are versatile porous materials that are established in chiral applications, but their instability under certain conditions in some cases requires the use of more stable porous materials such as carbons. In addition to their stability, porous carbon materials can be tailored for their ability to adsorb and catalytically activate different chemical compounds from the liquid and the gas phase. The difficulties imposed by the functionalization of carbons with chiral species were tackled in the past by carbonizing chiral ionic liquids (CILs) together with a template to create pores, which results in the entire body of a material that is built up from the precursor. To increase the atomic efficiency of ionic liquids for better economic utilization of CILs, the approach presented here is based on the formation of a composite between CIL-derived chiral carbon and a pristine carbon material obtained from carbohydrate precursors. Two novel enantioselective carbon composite materials are applied for the chiral recognition of molecules in the gas phase, as well as in solution. The enantiomeric ratio of the l-composite for phenylalanine from the solution was (L/D) = 8.4, and for 2-butanol from the gas phase, it was (S/R) = 1.3. The d-composite showed an opposite behavior, where the enantiomeric ratio for phenylalanine was (D/L) = 2.7, and for 2-butanol from the gas phase, it was (R/S) = 1.3.

## Introduction

Some of the principal concepts in the pharmaceutical and agricultural industry rely on the fact that biological activity is directly correlated with the chirality of a chemical compound. This places high demands on the production of enantiopure compounds, which is commonly accomplished by asymmetric synthesis or chiral separation and is often monitored by chiral sensors.^1,2^ The separation of enantiomers is based on their interactions with a chiral selector, which can be immobilized on the surface of the solid support. Alternatively, it can be added to the mobile phase (in chromatographic techniques) or the background electrolyte (in capillary electromigration techniques).^3,4^

When supports are used, it is beneficial to increase the surface area available for chiral recognition. This is commonly obtained by the utilization of porous materials.^5^ Silica is often the material of choice for these applications because it has well-defined surface chemistry, making it a good platform for covalent modifications by chiral linker moieties.^6−8^ In addition, it can show intrinsic chirality induced by imprinting or templating with chiral molecules.^9,10^ Metal–organic frameworks (MOFs)^11−13^ and chiral polymers^14−17^ have also been investigated as possible candidates for chiral separations due to the versatility of their synthetic pathways. However, the major drawback of these porous materials is their instability in extreme pH regions.^18^ Also, MOFs are in some cases moisture-sensitive and thus difficult to handle.^19,20^ This is an important factor in their application for chiral separations since the adsorbents have to be regenerated, which is difficult if the materials are thermally and chemically sensitive.^5^

A class of porous materials with superior thermal and chemical stability, as well as intrinsic electric conductivity, is porous carbon materials. They are well-established as adsorbents^21−23^ and thus have the possibility for utilization in chiral separations through classic chromatographic methods, the selectivity of which could be further controlled by an applied electric potential.^24−26^ Asymmetric (electro)catalysis and chiral sensing are further potential applications.^27,28^ In comparison to nanoporous carbons with random local atomic structure, allotropes of carbon with higher atomic order, such as carbon nanotubes (CNTs), graphene, or fullerene, have been more intensely studied for chirality-related applications.^29−31^ The likely reason is the uncomplicated uniform functionalization of their well-defined surface by covalent chemical coupling of functional groups. Nonetheless, these types of carbon materials are not intrinsically porous. Porosity, however, is an important property of materials used for adsorption, catalysis, and sensing in order to achieve high loading capacities and selectivity toward a given target molecule.^32,33^

In contrast, it remains rather difficult to chemically functionalize the heterogeneous surface of porous carbon materials with chiral groups. Therefore, several attempts in the synthesis of porous carbons directly from chiral precursors have been reported, mostly using templating approaches for the creation of pores and high carbonization yield.^34^ Materials already recognized as a suitable platform for carbon precursors are ionic liquids (ILs), as their cations or anions can easily be modified with chiral functions.^35,36^ The first studies reporting the synthesis of enantioselective porous carbons have also used chiral ILs, and eutectic salt melts or hard templates have been used to create porosity.^37−39^ However, the disadvantage of ILs as carbon precursors is that they are rather expensive, and their usage, especially when just carbonizing them, cannot always be justified by their excellent properties. Usually, ILs are carbonized together with a template to create pores, and in such a case, the entire body is built up from this precursor.

To maximize the atomic efficiency of the IL precursors and to ensure control over the porosity of the resulting carbon material, composite materials can be formed, combining two (or more) sets of properties in one material.^40−43^

The approach reported in the present study is the formation of stable composites consisting of a pristine porous carbon material and a chiral carbon coating derived from a chiral ionic liquid (CIL). Pristine carbon serves as a host material that has a well-defined pore architecture and a stable backbone, although its surface atomic functions are rather hydrophobic and heterogeneous. Because of the difficulties in chemical functionalization of the heterogeneous surface of porous carbon materials, it can be expected that the formation of homogeneous chiral coating is also challenging.^32,40,44^ Therefore, a layer of C_2_N-type polar coating is deposited on the surface of pristine carbon as a mediator, which readily interacts with the CIL. After low-temperature carbonization of the CIL coatings from two different enantiomers, two carbon composites with opposite chiral information are obtained. Their chiral recognition is investigated in the gas phase by the adsorption of chiral vapor and in solution by isothermal titration calorimetry with enantiopure titrants.

## Experimental Section

### Synthesis of Materials

#### Synthesis of C (CMK-3)

The hexagonal ordered silica template SBA-15 was synthesized by dissolving 33.4 g of the triblock copolymer Pluronic P123 (EO_20_PO_70_EO_20_, Sigma Aldrich) in 606 g of deionized water and 19.3 g of concentrated aqueous hydrochloric acid solution overnight at 35 °C in a 1000 mL polypropylene bottle under intense stirring. Then, 71.8 g of tetraethyl orthosilicate (TEOS, 98%, Sigma Aldrich) was added to the solution, and the mixture was stirred at 35 °C for another 24 h. The white suspension was then transferred to a Teflon-lined autoclave and hydrothermally treated at 130 °C for 24 h followed by filtration and washing with ∼1000 mL deionized water/ethanol (1:1 by volume). For complete removal of the structure-directing agent, SBA-15 was calcined at 550 °C for 5 h in a muffle furnace under an air atmosphere (heating rate, 60 °C h^–1^).^45^

Ordered mesoporous carbon CMK-3 was synthesized by impregnating 4 g of SBA-15 with a 20 mL aqueous solution of 5 g of sucrose to which was added 0.56 g of 96% sulfuric acid. Polymerization of the carbohydrate was achieved by heating the mixture to 100 °C for 6 h followed by subsequent heating to 160 °C for another 6 h. Complete infiltration of the template pores was achieved by repeating the procedure described above with a 20 mL aqueous solution of 3.2 g of sucrose to which was added 0.36 g of 96% sulfuric acid, again followed by heating to 100 and 160 °C. Carbonization was carried out under a flowing N_2_ atmosphere in a horizontal tubular furnace. The material was heated to 900 °C (heating rate, 150 °C h^–1^) and dwelled for 2 h. Silica removal was achieved by refluxing the carbonized composite material in sodium hydroxide solution (400 mL, 5 mol L^–1^) overnight. After filtration and washing with large amounts of ethanol, the CMK-3 material was dried at 60 °C.^46^

#### Synthesis of the C_2_N/C Composite

The C_2_N-type coating on C carbon was synthesized by wet impregnation of 200 mg of C with the solution of 267 mg of hexaazatriphenylene-hexacarbonitrile (HAT-CN)^47^ in 0.4 mL of dimethylformamide. After drying overnight at 70 °C under vacuum, the material was heated to 550 °C (heating rate, 240 °C h^–1^) and dwelled for 2 h.

#### Synthesis of l- and d-Composites

The chiral coating on the C_2_N/C composite was synthesized by wet impregnation of 179 mg of the C_2_N/C composite with the solution of 230 μL of the *N*,*N*-dimethyl-l-proline methyl ester bis(trifluoromethylsulfonyl)imide (l-Pro-TFSI) chiral ionic liquid in 0.46 mL of dimethylformamide. The same procedure was followed for the d-enantiomer. After drying overnight at 70 °C under vacuum, the material was heated to 500 °C (heating rate, 240 °C h^–1^) and dwelled for 2 h.

### Characterization of Materials

#### Physisorption Measurements

Before the physisorption measurements, the samples were outgassed under vacuum at 150 °C for 20 h. N_2_ physisorption experiments were carried out at −196 °C on a Quadrasorb apparatus (Quantachrome Instruments, USA). Specific surface areas (SSA) of the materials are calculated using the multipoint Brunauer–Emmett–Teller (BET) model in the relative pressure range of 0.05–0.2. The total pore volumes (*V*_t_) were determined at *p*/*p*_0_ = 0.99. The pore size distributions are calculated using a quenched-solid density functional theory (QSDFT) method for nitrogen on carbon with slit/cylindrical/spherical pores at −196 °C, adsorption branch kernel, integrated into QuadraWin 5.11 analysis software (Quantachrome). Micropore volumes (*V*_micro_) are calculated using the DFT method from the cumulative pore volumes at a diameter of 2 nm. CO_2_ physisorption experiments were carried out at 0 °C on the Quadrasorb apparatus to investigate the pores with a diameter smaller than 1.5 nm. Corresponding PSDs were calculated by the nonlocal density functional theory (NLDFT) method for CO_2_ adsorbed on carbon at 0 °C. Water and chiral gas physisorption measurements were performed using (*S*)-(+)-2-butanol or (*R*)-(−)-2-butanol at 25 °C (sample weight, ∼50 mg) on a Quantachrome Autosorb IQ apparatus.

#### Scanning Electron Microscopy

SEM was carried out on a LEO 1550-Gemini microscope operating at 3.00 kV. The samples were coated with a few nm thin platinum layer via sputtering to increase the surface conductivity.

#### Energy-Dispersive X-ray Spectroscopy

EDX investigations were conducted using a Link ISIS-300 system (Oxford Microanalysis Group) equipped with a Si(Li) detector and an energy resolution of 133 eV.

#### Thermogravimetric Analyses

TGA were performed using a thermo-microbalance TG 209 F1 Libra (Netzsch, Selb, Germany). A platinum crucible was used for the measurements of 10 ± 1 mg of samples in synthetic air flow. The sample was heated to 1000 °C with a heating rate of 10 °C min^–1^. The data were recorded and analyzed by the Proteus (6.0.0) software package.

#### Transmission Electron Microscopy

TEM was carried out on a Zeiss912 Omega operated at 120 kV. Prior to analysis, the samples were dispersed in ethanol and sonicated for 5 min. Several droplets of dispersions were cast onto TEM copper grids with a holey carbon film and dried at room temperature.

#### Powder X-ray Diffraction

PXRD patterns were recorded on a Bruker D8 Advance diffractometer equipped with a scintillation counter detector using Cu Kα radiation (λ = 0.1518 nm) in the 2θ range of 360° with a step size of 0.02° and a counting time of 1 s per step.

#### Elemental Analysis

C/H/N/S elemental analysis (EA) was accomplished as a combustion analysis using a Vario Micro device.

#### Raman Spectroscopy

Raman spectra were recorded using a Witec Raman microscope operating with an objective (Nikon, 10×/0.25, ∞/– WD 6.1) and an excitation wavelength of 532 nm with an intensity of 3.5 mW and accumulations of 100 scans with 10 s per scan. Deconvolution of the spectra was performed by assuming mixed Gaussian/Lorentzian peaks to describe both the main D- and G-bands and the two bands with lower intensity, A and D^2^, positioned at 1500 and 1170 cm^–1^, respectively. The fit was performed using OriginPro 2019. The parameters retained were the wavenumber of the G-band (statistical analysis performed on 4 spectra per sample) and the ratio of the peak heights (*I*_D_/*I*_G_).

#### Isothermal Titration Calorimetry

ITC measurements were performed using a VP-ITC microcalorimeter from MicroCal (Northampton, USA). Two identical spherical cells, a reference cell and a sample cell, both with a volume of 1.442 mL, were enclosed in an adiabatic jacket. The working cell was filled with an aqueous dispersion of the sample, and the reference cell was filled with water. The titrant (60 mM l-phenylalanine or 60 mM d-phenylalanine) was injected stepwise into the working cell with a syringe with the total volume of 288 μL. The sample cell was constantly stirred at a stirring rate of 307 rpm. The measurements were performed at a constant temperature of 25 °C. Small aliquots of the titrant (5 μL) were injected into the solution of the working cell. The first injection was set to a volume of 2 μL because of the possible dilution during the equilibration time preceding the measurement, and therefore, the first injection was ignored in the analysis of the data. Spacing between the injections was set to 300 s. Data analysis was performed using Origin software provided by MicroCal.

## Results and Discussion

**Scheme 1:**
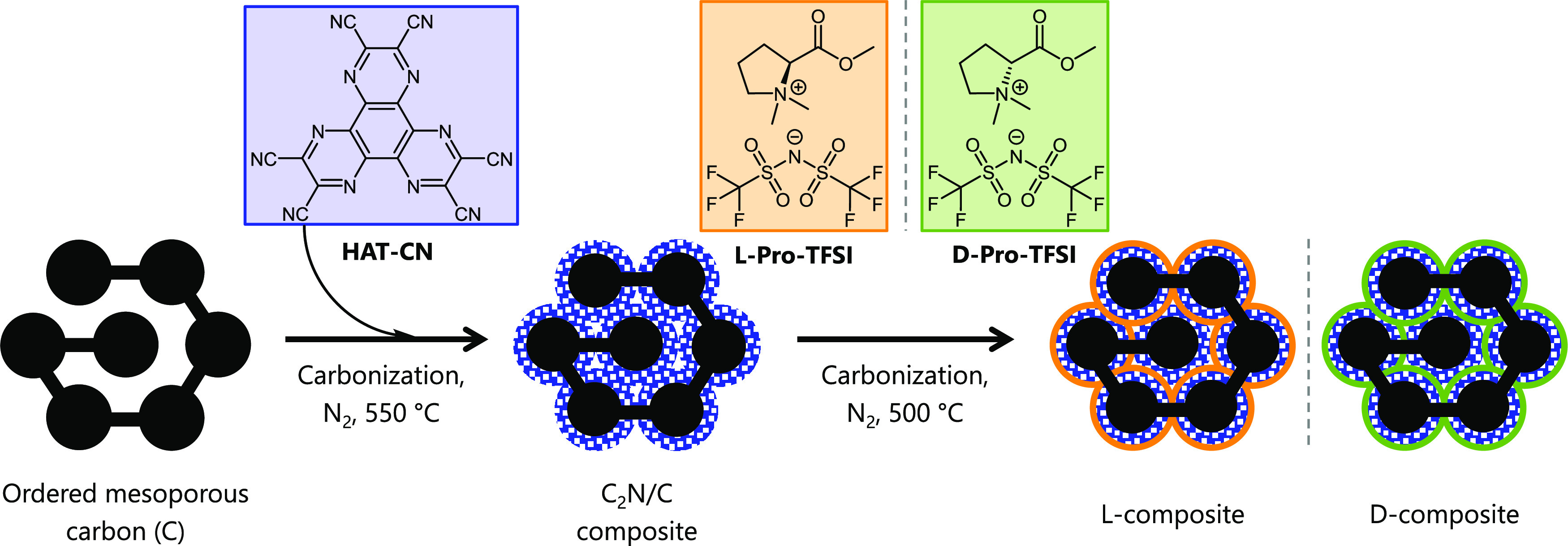
The Synthesis Procedure of the C_2_N/C Composite and l-/d-Composites

Carbon composite materials with chiral surfaces were synthesized by coating porous carbon with a chiral ionic liquid and its subsequent carbonization. Ordered mesoporous carbon (CMK-3, in further text denoted as C) was obtained by impregnation of a silica hard template (SBA-15) with sucrose followed by its carbonization and template removal.^45,46^ For providing more homogeneous and polar surface chemistry, C was coated with hexaazatriphenylene-hexacarbonitrile (HAT-CN) (in the blue rectangle in Scheme 1), which was then heated to 550 °C under nitrogen flow.^47^ The controlled condensation of this nitrogen-rich organic molecule yields a nitrogen-doped carbonaceous network with almost perfect C_2_N-type stoichiometry.^48^ The resulting C_2_N/C composite was subsequently coated with the *N*,*N*-dimethyl-l-proline methyl ester bis(trifluoromethylsulfonyl)imide (l-Pro-TFSI) chiral ionic liquid (CIL) (in the orange rectangle in Scheme 1) or its d-enantiomer (in the green rectangle in Scheme 1). l-Pro-TFSI and d-Pro-TFSI CILs were synthesized by a previously reported procedure.^37,49^ After the carbonization of the CIL coating on 500 °C in an inert atmosphere, final composite materials were obtained, labeled as the “l-composite” and “d-composite”.

**Figure 1:**
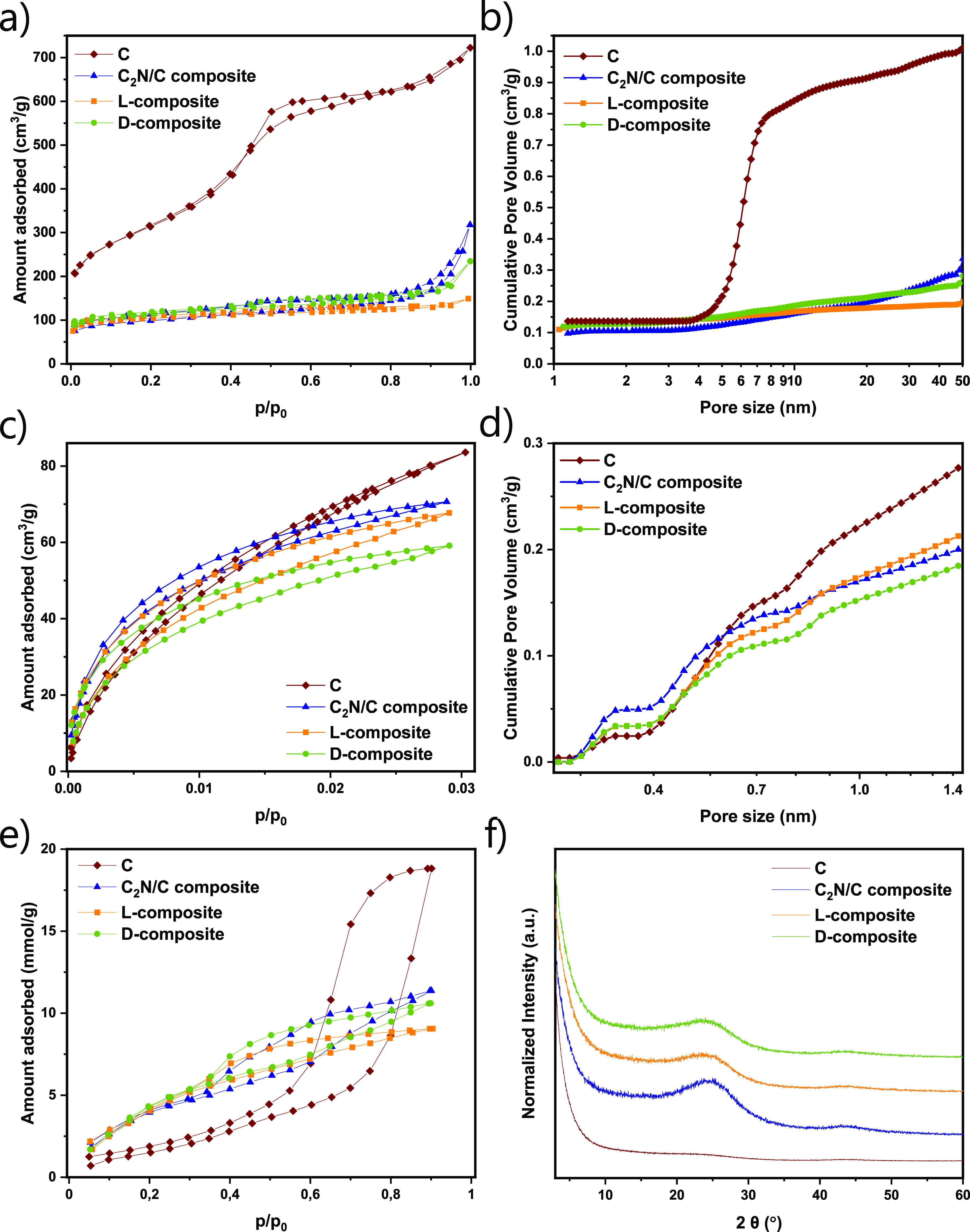
(a) N_2_ physisorption isotherms (at −196 °C) with the corresponding (b) semilogarithmic cumulative pore size distribution plots calculated with QSDFT, (c) CO_2_ physisorption isotherms (at 0 °C) with the corresponding (d) semilogarithmic cumulative pore size distribution plots calculated with NLDFT, (e) water vapor physisorption isotherms (at 25 °C), and (f) XRD patterns of C, the C_2_N/C composite, and l- and d-composites.

N_2_ physisorption experiments (−196 °C) were carried out to investigate the pore structures of the carbon and the composite materials (Figure 1a,b). C displays a type IV(a) isotherm according to IUPAC classification, with an H2(a) hysteresis loop in the relative pressure range of 0.45–0.75, which is typical for ordered mesoporous adsorbents.^50^ This material was chosen as a support material for the formation of composites due to its ordered structure with a large amount of well-interconnected mesopores. This pore architecture of C enables infiltration of the HAT-CN precursor into the body of pristine carbon. Upon the formation of the C_2_N/C composite, the pores of C carbon were filled with the C_2_N material. This leads to a substantial decrease in the porosity. The C_2_N-type material obtained through condensation of the HAT-CN precursor is entirely microporous,^48^ and thus, it is expected that the resulting composite contains a considerable amount of micropores as well. The C_2_N/C composite and l- and d-composites exhibit a combination of type I and type II isotherms, as typical for mainly microporous solids with a certain amount of external porosity, deduced from the nitrogen uptake above a relative pressure of 0.95. All three composite materials show a minor hysteresis, most likely originating from the remaining mesopores of the C support material.

TFSI is a large anion that is commonly believed to act as a sacrificial template in the formation of porous carbons from ionic liquids.^51,52^ The carbonaceous materials derived from l- and d-Pro-TFSI are porous (Figure S1, Supporting Information, CO_2_ pore volume equals 0.25 cm^3^ g^–1^). It is therefore reasonable that the nitrogen uptake of l- and d-composites remains comparable to the parent C_2_N/C composite.

For the analysis of narrow micropores, CO_2_ physisorption (0 °C) experiments were carried out (Figure 1c,d). The convex shape of the isotherms of the composite materials together with the presence of hysteresis reveals a high affinity to CO_2_ at low relative pressure caused by the high content of nitrogen atoms in the composites providing specific binding sites for the adsorption. The isotherm of C is rather linear, without pronounced hysteresis. The affinity of the composites toward CO_2_ and their uptake are comparable, as it is the case for N_2_.

The pore size distributions (PSDs) of the materials were analyzed by quenched-solid density functional theory (QSDFT, adsorption branch kernel) for N_2_ adsorbed on carbon with a slit/cylindrical/spherical pore shape and nonlocal DFT analysis of CO_2_ physisorption measurements (Figure 1b,d and Table 1). C has a high mesopore volume of 0.86 cm^3^ g^–1^ and a narrow PSD centered around a diameter of 6 nm. The volume of pores with a diameter below 1.5 nm, determined by CO_2_ physisorption, is 0.21 and 0.18 cm^3^ g^–1^, for l- and d-composites, respectively. The volume of ultramicropores (diameter < 0.7 nm) is significant for both composites and equals 0.13 and 0.11 cm^3^ g^–1^, for l- and d-composites, respectively. l- and d-composite materials exhibit comparable (multipoint) Brunauer–Emmett–Teller specific surface areas (SSA_BET_) of 395 and 418 m^2^ g^–1^, respectively.

**Table 1:** Gas Adsorption, EA and EDX Data Summary (in wt %), and *I*_D_/*I*_G_ Obtained from Raman Spectra of C, the C_2_N/C Composite, and l- and d-Composites^a^

						C		N				
sample	SSA_BET_ [m^2^ g^–1^]	*V*_meso_ [cm^3^ g^–1^]	*V*_N2 (<2 nm)_ [cm^3^ g^–1^]	*V*_CO2 (<1.5 nm)_ [cm^3^ g^–1^]	*V*_CO2 (<0.7 nm)_ [cm^3^ g^–1^]	EA	EDX	EA	EDX	*H*_EA_	*S*_EA_	*I*_D_/*I*_G_
C	1122	0.86	0.14	0.03	0.02	81.4		0.2		1.4	1.0	1.00
C_2_N/C composite	356	0.22	0.10	0.20	0.14	65.7		18.0		1.6	0.3	1.02
l-composite	395	0.08	0.13	0.21	0.13	67.8	73.9	15.7	22.9	1.6	0.6	1.24
d-composite	418	0.14	0.13	0.18	0.11	67.9	77.1	16.4	19.7	1.5	0.2	1.23

a In addition to the elements in the table, the content of O_EDX_ in the l-composite is 3.2 wt %, and in the d-composite, it is 3.1 wt %.

Water vapor adsorption (25 °C) was measured to compare the surface polarity of these materials (Figure 1e). C exhibits a type V isotherm, typical for adsorption of water on rather hydrophobic micro- and mesoporous materials. As a consequence of their high nitrogen content of ∼16 wt % as determined by C/H/N/S elemental analysis (EA) discussed below, the onset point of water adsorption on composite materials is at low relative pressures. Below a *p*/*p*_0_ of 0.4, specific interactions between water and nitrogen functionalities are responsible for a steep increase in the water uptake. l- and d-composites have an identical shape of the isotherms below a relative pressure of 0.6, where such surface–water interactions are dominant. The minor difference above this pressure with a slightly higher total uptake for the d-composite is due to the adsorption in the wider micropores and narrow mesopores, and it follows the behavior already observed in N_2_ physisorption.

X-ray powder diffraction (XRD) measurements of C and the composite materials show patterns without sharp peaks that are typical for disordered porous carbon materials without inorganic impurities (Figure 1f). All composite materials apparently contain nanoscaled domains with graphite-like carbon stacking and thus reveal broad (002) and (101) carbon peaks at ≈25 and ≈44° 2θ. l- and d-composites have a slightly shifted (002) peak to lower angles in comparison to the C_2_N/C composite, implying that the carbonaceous layer obtained from the CIL might have a larger distance between graphitic layers. The absence of obvious carbon peaks and the high scattering at low angles in the C sample could be ascribed to its high porosity.

**Figure 2:**
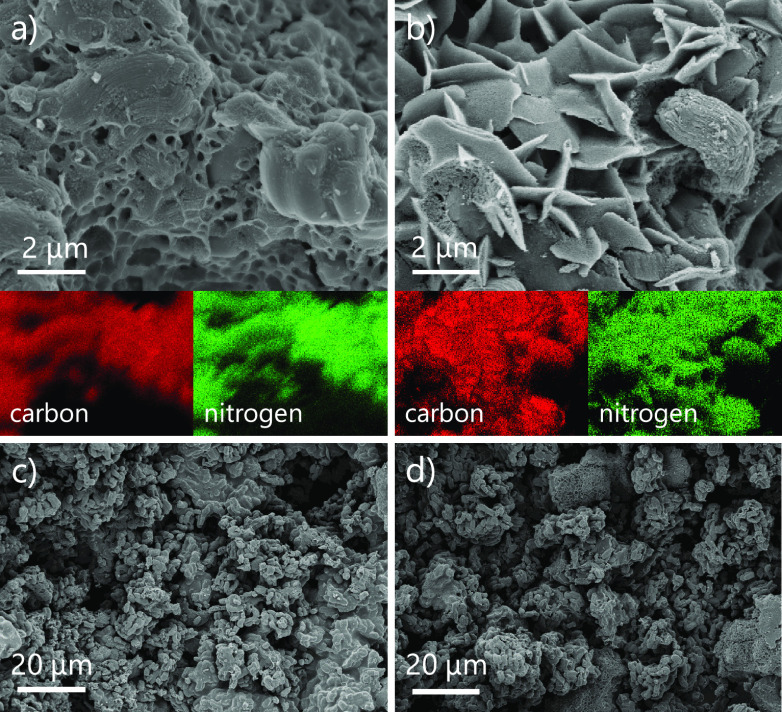
SEM images with EDX elemental mapping of (a,c) l-composite and (b,d) d-composite.

Scanning electron microscopy (SEM) images of l- and d-composites show that their morphologies are very similar (Figure 2). The images reveal cylindrical shapes originating from the C support material. These structures often have a coating made of a material with a smooth surface, which can be assigned to C_2_N since it is known that it exhibits this kind of morphology.^48^ Additional features observed in both composites are sponge- and shell-like structures, arising from the carbonized CIL and C_2_N material.

The results of EA illustrate the process of composite formation, through their bulk elemental composition values (Table 1). From the predominant carbon content and with almost no nitrogen present in C, the formation of the C_2_N/C composite results in the decreased carbon content of 65.7 wt %, while the nitrogen content increases to 18.0 wt %. Upon deposition of the CIL-derived carbon layer, the carbon content slightly increases, while the nitrogen content decreases. l- and d-composites have almost identical chemical compositions, with 67.8 and 67.9 wt % C and 15.7 and 16.4 wt % N in l- and d-composites, respectively. Energy-dispersive X-ray (EDX) spectroscopy of l- and d-composites presents slightly higher values for both carbon and nitrogen and around 3 wt % O. This can be ascribed to several factors, including the polarity of the materials, which makes them good adsorbents for moisture and carbon dioxide from air, which will constitute a part of the initial mass measured in EA.^53^ Another reason can also be that in EDX, the sum of the contents of elements is normalized to 100%. Therefore, it makes sense to compare the carbon/nitrogen mass ratios as determined by EA (4.32 for the l-composite and 4.14 for the d-composite) and EDX (3.23 for the l-composite and 3.91 for the d-composite), which are very close to each other for both composites. EDX elemental mapping patterns display a homogeneous distribution of carbon and nitrogen in l- and d-composites (Figure 2a,b). This shows that the formation of the C_2_N/C composite offers a homogeneous surface for the attachment of the ionic liquid and that the carbonized CILs are evenly distributed on the surface of the support. Thermogravimetric analysis of the l- and d-composites under synthetic air reveals their increased oxidation stability in comparison to their host carbon material C (Figure S2, Supporting Information). This can be ascribed to their lower porosity and high nitrogen content. The insertion of heteroatoms into the carbon backbone most often has a stabilizing effect on carbon materials.

Transmission electron microscopy (TEM) images of l- and d-composites also demonstrate the ordered mesoporous system of the C host, together with typical microporous amorphous carbon morphology. The C_2_N/C composite shows similar microporous morphology (Figure S3, Supporting Information).

**Figure 3:**
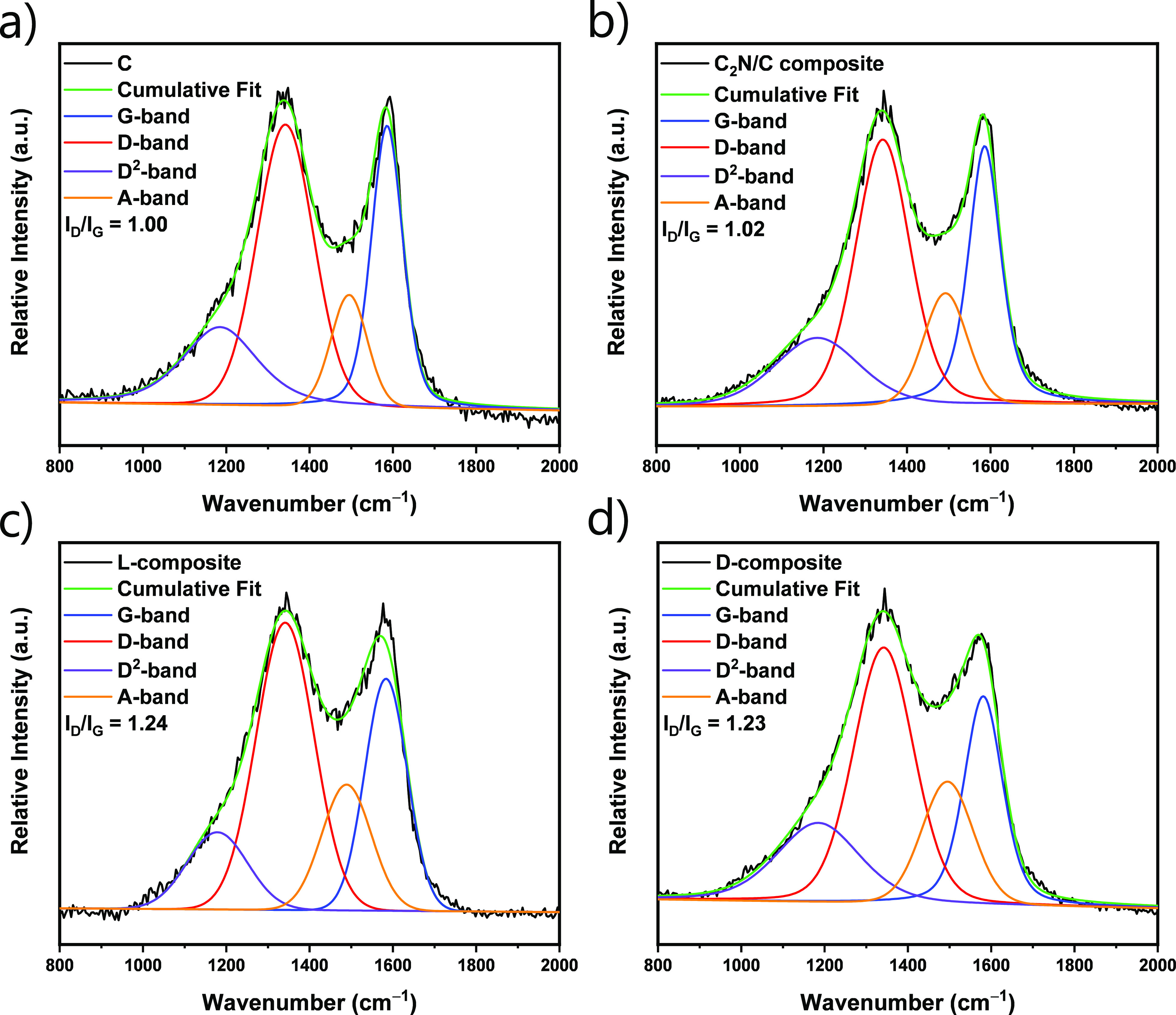
Deconvoluted Raman spectra of (a) C, (b) C_2_N/C composite, (c) l-composite, and (d) d-composite.

Raman spectra of the prepared materials were fitted by using a 4-band model with mixed Gaussian/Lorentzian peaks (Figure 3).^54^ C carbon shows a spectrum as expected for disordered carbon materials, with the D-band at ∼1340 cm^–1^ and the G-band at ∼1595 cm^–1^. The D-band (“disordered”) originates from the breathing mode of sp^2^-hybridized carbon atoms in aromatic rings neighbored by a defect. The G-band (graphite-like) originates from vibrations of all sp^2^ carbons organized in chains or rings. The peak intensity ratio of D- and G-bands (*I*_D_/*I*_G_) states the degree of carbon ordering in porous carbon materials.^55,56^ For the composite materials, the standard model for the interpretation and analysis of Raman spectra for porous carbons cannot be directly implemented because nitrogen doping causes vibrational dissymmetry. However, certain conclusions about their structure can indeed be drawn through a comparison with the nondoped support material. The values of *I*_D_/*I*_G_ for the C and C_2_N/C composite are almost identical (1.00 and 1.02 cm^–1^, respectively), which indicates a comparably high degree of aromatization and that the Raman signal of C remains almost unaffected by coating with C_2_N. This is probably due to a high level of ordering during the controlled condensation of HAT-CN into the C_2_N material. l- and d-composites have almost identical *I*_D_/*I*_G_ values of 1.24 and 1.23 cm^–1^, respectively, suggesting a comparable nanostructure in accordance to gas physisorption, EA, and microscopy analysis. Furthermore, the higher contribution of the A-band in the composite materials, in comparison to the C and C_2_N/C composite can be ascribed to the low-temperature carbonization of CIL coating, which does not undergo a controlled carbonization pathway like the C_2_N material and results in a rather disordered structure.

Structural and chemical characterization of l- and d-composites reveals their almost identical structure, including porosity, morphology, surface, and bulk chemistry. They are, however, synthesized from two different enantiopure ionic liquids, and it can be expected that they will interact differently with the enantiomers of the same compound if chiral information of the CIL could be transferred to the composites. For further investigation of this hypothesis, isothermal titration calorimetry (ITC) was employed.^57−59^ This technique offers information on thermodynamic parameters of interactions in the solution and is thus convenient for measuring differences in the interactions between both composites. In the experiments, equal aliquots of one enantiomer of phenylalanine (Phe) were titrated into the dispersion of the l- or d-composite, and the heat response upon injection is monitored.

**Figure 4:**
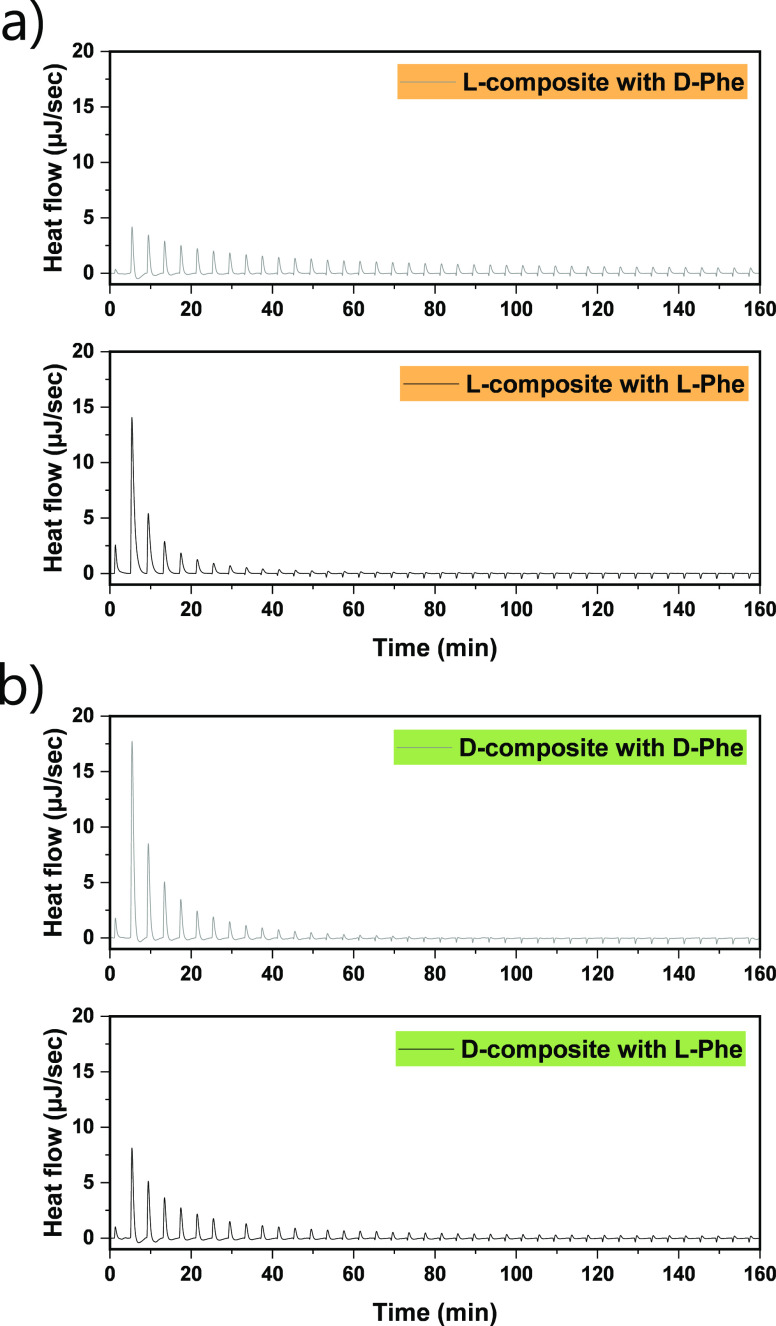
Raw isothermal titration calorimetry data from titration of (a) l-composite and (b) d-composite with d-phenylalanine (upper panels, gray line) and l-phenylalanine (lower panels, black line).

Absorbed heat upon the addition of l-Phe in the dispersion of the l-composite is higher than heat upon the addition of d-Phe in the same material (Figure 4a). At the beginning of the titration, the pores of the composite are filled with water, and the first molecules of the titrant therefore induce the highest heat response. First injections are responsible for specific interactions between the porous composite with the chiral surface and a chiral molecule. The first injection of l-Phe into the dispersion of the l-composite causes 8.4 times higher heat flow than the interaction with d-Phe, reaching 7.3 kJ per mole of the amino acid (Figure S4a, Supporting Information). Upon further addition of l-Phe, this difference substantially decreases, yielding 2.8 and 1.6 times more heat in the second and third injection, respectively, in comparison to titration with d-Phe. As the solution becomes saturated with amino acid, the nonspecific interactions increase, and therefore, not only the heat responses of both samples but also their relative difference decreases. Due to the complex processes happening throughout the titration, including desolvation, dilution, desorption of solvent from the pores, and adsorption of a chiral molecule on the surface of a material, and due to the very hydrophilic properties of the C_2_N coating, it is difficult to precisely determine the single contributions of each factor, especially after the first couple of injections. The interactions upon titration of the d-composite reveal the opposite trend for the l-composite, with higher heat flow upon injection with d-Phe than with l-Phe (Figure 4b). This corresponds to 2.7, 2.0, and 1.4 times more heat absorbed after the first, second, and third injection with d-Phe in comparison to l-Phe (Figure S4b, Supporting Information). Similarly to the l-composite, these differences become less pronounced when the concentration of amino acid in the solution increases. Based on this, the enantiomeric ratio of the l-composite for phenylalanine is (L/D) = 8.4, and for the d-composite, it is (D/L) = 2.7. ITC control experiments were conducted by titrating the C_2_N/C composite with enantiopure solutions of phenylalanine (Figure S5, Supporting Information). These results reveal only a minor difference in heat flow signals between two different enantiomers of a titrant. In addition, the strength of these signals is relatively low in comparison to the interactions between chiral composites and chiral solutions. Therefore, it can be concluded that indeed, the chiral coating on the C_2_N/C composite is responsible for specific interactions between these materials and enantiopure solutions.

The adsorption capacity of these composites is, however, relatively limited due to their narrow micropores, which are partially also filled with the solvent molecules. To decouple the adsorption process from the influence of the solvent, chiral recognition of the composites was also investigated by the physisorption of chiral vapor, thus measuring in a 2-compound system. The probe adsorbates used in the experiments are the enantiomerically pure forms of 2-butanol, namely, (*R*)-(−)-2-butanol and (*S*)-(+)-2-butanol. Both enantiomers were adsorbed on the activated (outgassed) composites at 25 °C.

**Figure 5:**
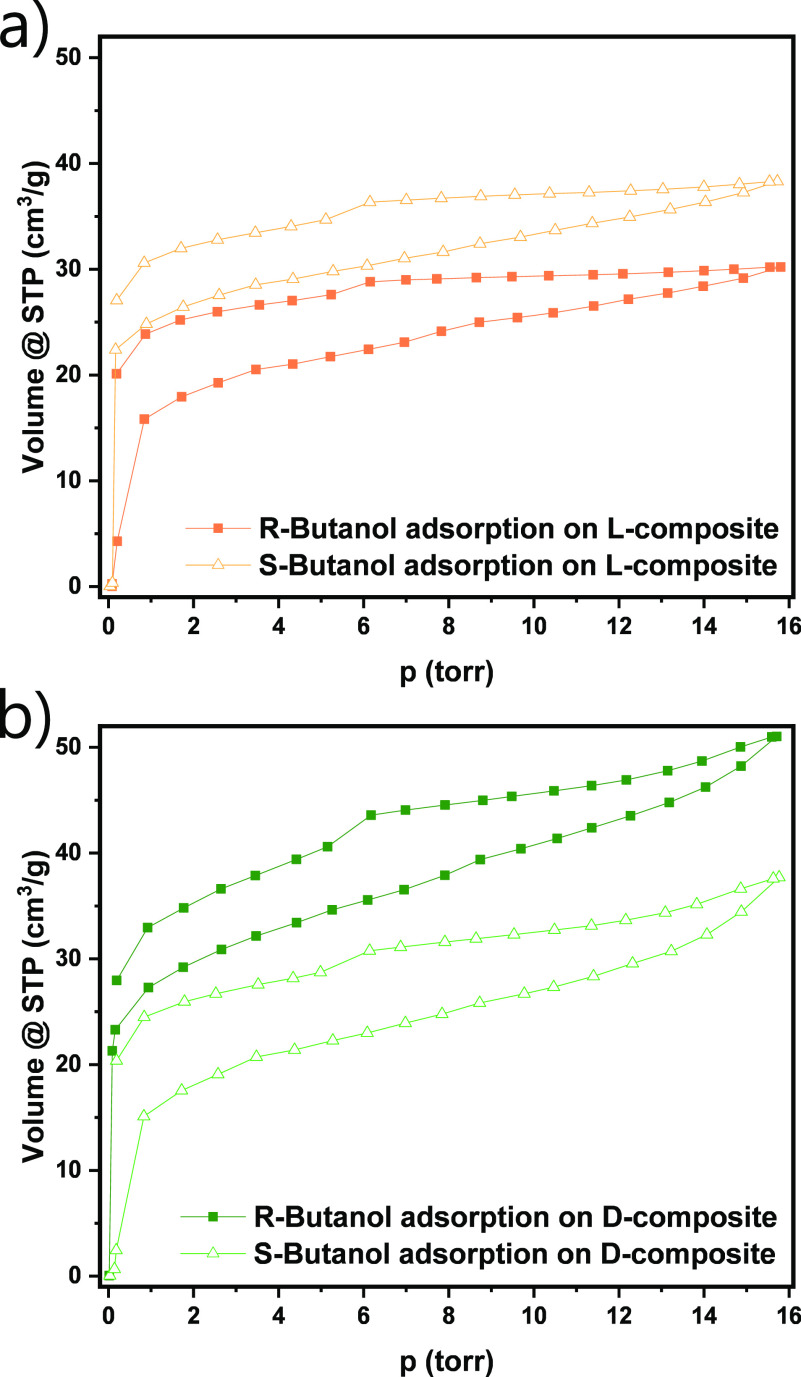
Chiral vapor adsorption isotherms (25 °C) of (a) l-composite and (b) d-composite with *R*-butanol (solid squares) and *S*-butanol (hollow triangles).

Adsorption of *S*-butanol on the l-composite has considerably higher vapor uptake than adsorption of *R*-butanol, throughout the entire range of applied pressures (Figure 5a). The trend is opposite for adsorption on the d-composite (Figure 5b). Similarly to ITC, it is expected that the specific chiral interactions between the composite material and the adsorbate take place predominantly in the low-pressure region when the chiral centers in the material are still accessible and adsorbent–adsorbate interactions are dominant. The (ultra)micropore structures of l- and d-composites are almost identical, as it was shown with N_2_ and CO_2_ physisorption measurements (Figure 1). Thus, it is not surprising that the shape of butanol adsorption isotherms in the low-pressure region is comparable for both materials and both enantiomers of butanol. However, different uptakes of enantiomers of butanol can be assigned to chiral recognition taking place between the vapor and the pore walls of the composite. The adsorbed volumes are also in a comparable range. For instance, at 1.7 Torr (corresponds to a relative pressure of 0.09), the l-composite adsorbed 17.9 cm^3^ g^–1^ of *R*-butanol and 26.4 cm^3^ g^–1^ of *S*-butanol, whereas the d-composite adsorbed 29.2 cm^3^ g^–1^ of *R*-butanol and 17.5 cm^3^ g^–1^ of *S*-butanol. In the higher-pressure region and close to the saturation pressure of butanol, the shape of the isotherms is similar to N_2_ and water vapor isotherms, with an obvious rise in the uptake of the adsorbate on the d-composite. Considering that in this range, it comes to multilayer adsorption, it can be assumed that chiral recognition no longer has a significant influence on the resulting vapor uptake, and the slope of the isotherms remains comparable for both composites. The enantiomeric ratio expressed on 1.7 Torr of the l-composite for butanol is (S/R) = 1.5, and for the d-composite, it is (R/S) = 1.7. If the enantiomeric ratio is expressed at the highest uptake of butanol, then it equals an S/R of 1.3 for the l-composite and an R/S of 1.3 for the d-composite.

## Conclusions

A template-free approach that offers better economic utilization of chiral precursors and separates the creation of pores from the creation of chiral carbon has been successfully applied here for the synthesis of chiral carbon composite materials. Carbon composites with opposite chiral surface functionalities have been synthesized by the coating of pristine carbon with a chiral ionic liquid and its subsequent low-temperature carbonization. A layer of the C_2_N-type material between pristine carbon and the chiral coating served as a polar mediator to ensure more homogeneous interaction with the ionic liquid. Resulting l- and d-composites are mainly microporous materials that have almost identical porosity, morphology, and bulk and surface chemistry. They, however, show an opposite behavior in the interactions with enantiopure compounds from the solution and the gas phase due to specific chemical interactions, which is a property that can enable potential application in the separation of enantiomers. The enantiomeric ratio of the l-composite for phenylalanine from the solution was (L/D) = 8.4, and for 2-butanol from the gas phase, it was (S/R) = 1.3. The d-composite showed an opposite behavior, where the enantiomeric ratio for phenylalanine was (D/L) = 2.7, and for 2-butanol from the gas phase, it was (R/S) = 1.3. The creation of nanocomposites between pristine carbon and heteroatom-doped carbon that contains chiral information has been presented as a viable solution toward establishing carbonaceous materials with an enantioselective surface.

## Supporting Information Available

CO_2_ physisorption isotherm of the carbonized chiral ionic liquid; TEM images of composite materials; TGA results of C, l-, and d-composites under synthetic air; integrated ITC data; and ITC data of the control experiment from titration of the C_2_N/C composite (PDF)
