# Supplementary material for: Toward Efficient Synthesis of Porous All-Carbon-Based Nanocomposites for Enantiospecific Separation
Source: ACS Appl Mater Interfaces. 2021 May 12;13(20):24228–37. doi: 10.1021/acsami.1c02673 (PMC8289191; doi:10.1021/acsami.1c02673)
Supplement: Supplementary file 1 — am1c02673_si_001.pdf [file am1c02673_si_001.pdf]

Supporting Information

Toward Efficient Synthesis of Porous All-Carbon-Based Nanocomposites for Enantiospecific Separation

Milena Perovic,[a] Sapir Shekef Aloni,[b] Wuyong Zhang,[a] Yitzhak Mastai,[b] Markus Antonietti,[a] and Martin Oschatz*[a,c]*

[a] Dr. M. Perovic, W. Zhang, Prof. M. Antonietti, Prof. M. Oschatz

Department of Colloid Chemistry, Max-Planck Institute of Colloids and Interfaces, Am Mühlenberg 1, 14476 Potsdam, Germany

[b] S. Shekef Aloni, Prof. Y. Mastai

Department of Chemistry and the Institute of Nanotechnology, Bar-Ilan University, Ramat-Gan 5290002, Israel

[c] Prof. M. Oschatz

Friedrich-Schiller-University Jena, Institute for Technical Chemistry and Environmental Chemistry, Center for Energy and Environmental Chemistry Jena (CEEC Jena), Philosophenweg 7a, 07743 Jena, Germany

E-mail: martin.oschatz@uni-jena.de

Supplementary Figures

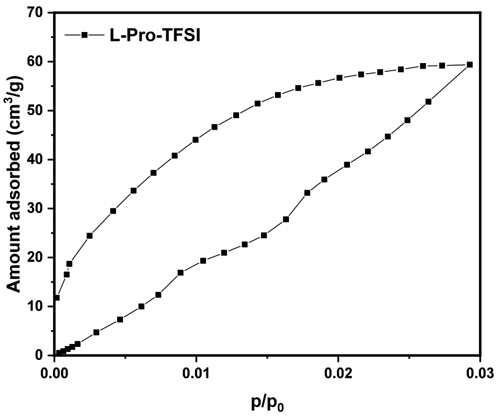

Figure S1. *CO_2 physisorption isotherm (at 0 °C) of L-Pro-TFSI chiral ionic liquid carbonized at 400 °C. Measurement performed on a sample m<10 mg.*

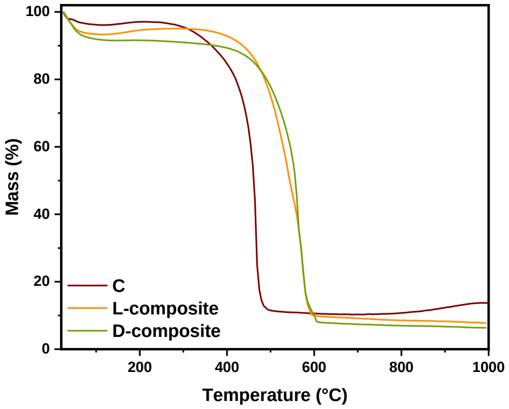

Figure S2. *Thermogravimetric analysis (TGA) results of C, and L-, and D-composites measured under synthetic air with a heating rate of 10 °C min^{-1}.*

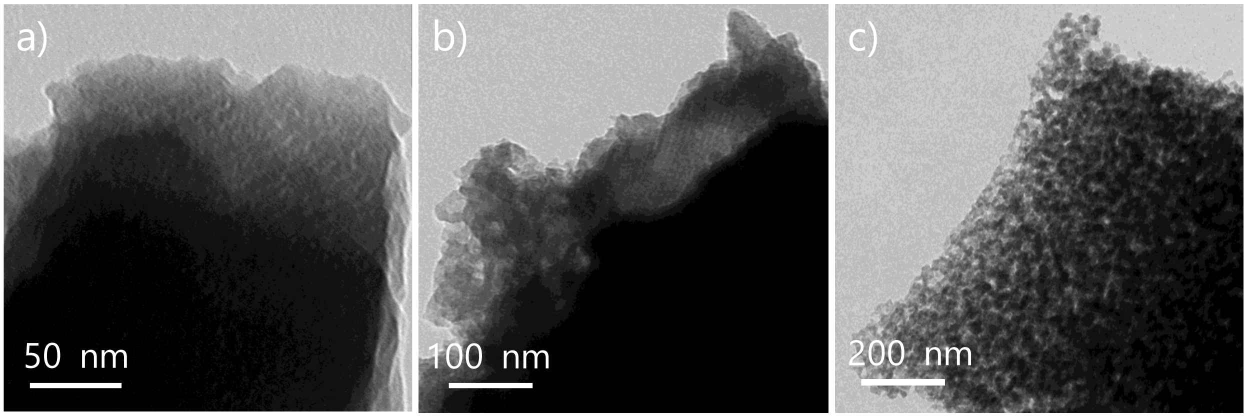

Figure S3. *TEM images of a) L-composite, b) D-composite, and c) C_2N/C composite.*

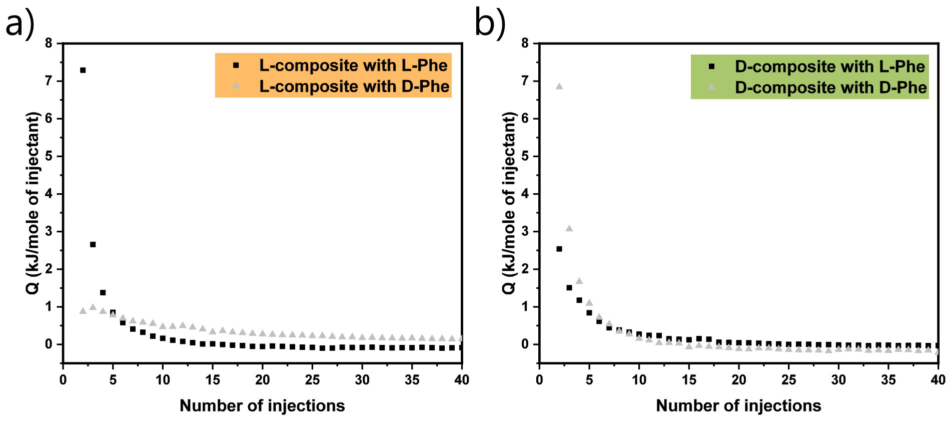

Figure S4. *Integrated ITC data of the titration of a) L-composite, and b) D-composite.*

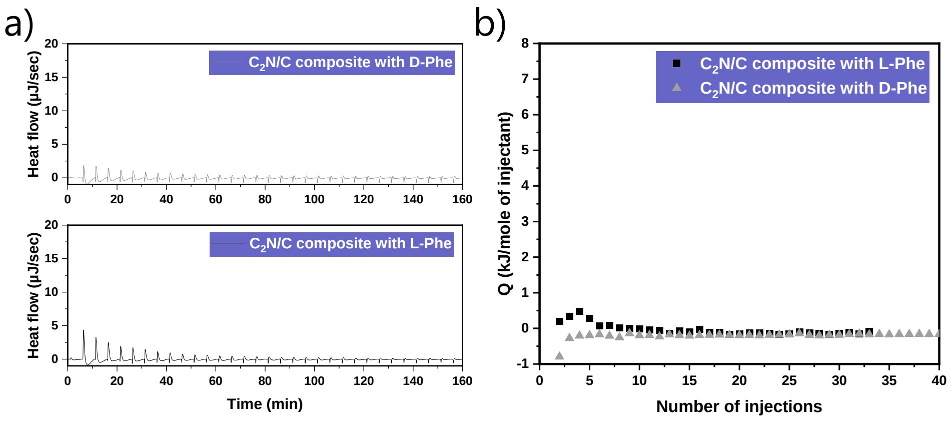

Figure S5. *a) Raw isothermal titration calorimetry data from titration of C_2N/C composite with D-Phenylalanine (upper panel, gray line), and L-Phenylalanine (lower panel, black line), and b) integrated ITC data of the titration of C_2N/C composite.*
